# [Retracted] Deoxypodophyllotoxin inhibits cell viability and invasion by blocking the PI3K/Akt signaling pathway in human glioblastoma cells

**DOI:** 10.3892/or.2023.8605

**Published:** 2023-07-24

**Authors:** Wei Wang, Wei Gao, Luyang Zhang, Dongyong Zhang, Zilong Zhao, Yijun Bao

Oncol Rep 41: 2453–2463, 2019; DOI: 10.3892/or.2019.7016

Following the publication of the above article and a corrigendum that was published to address issues of duplicated data panels in Fig. 4 (doi: 10.3892/or.2023.8484), a concerned reader has drawn to the Editor's attention that Fig. 3B also contains a matching pair of identical flow cytometry scatterplots where the results from different experiments were intended to have been portrayed, and certain of the western blotting data shown in Fig. 3C are strikingly similar to data that had appeared in Figs. 2 and 3 in a previously published paper written by different authors at different research institutes [Tian F, Ding D and Li D: Fangchinoline targets PI3K and suppresses PI3K/AKT signaling pathway in SGC7901 cells. Int J Oncol 46: 2355-2363, 2015].

In view of the fact that certain of the data in the above article had already appeared in a previously published paper, and given the large number of apparently overlapping data panels identified in several of the figures, the Editor of *Oncology Reports* has decided that this paper should be retracted from the publication. After having been in contact with the authors, they accepted the decision to retract the paper. The Editor apologizes to the readership for any inconvenience caused.

